# 

**Published:** 1909-01

**Authors:** 


### CONTENTS—JANUARY, 1909.—VOL. XXIV, NO. 7.

ORIGINAL ARTICLES—

Medical Politics in Epigram and Otherwise. By G. Frank Lydston, M. D., Chicago .......... 275

Additional Provision for the Insane. By F. S. White, Terrell, Texas .......... 290

EDITORIAL DEPARTMENT—

Chronicles of the Octopus .......... 296

The Fly in the Ointment .......... 301

EDITORIALETS .......... 302

CORRESPONDENCE .......... 304

